# Selection of patients with severe pelvic fracture for early angiography remains controversial

**DOI:** 10.1186/1757-7241-17-62

**Published:** 2009-11-29

**Authors:** Igor Jeroukhimov, Itamar Ashkenazi, Boris Kessel, Vladimir Gaziants, Amir Peer, Alexander Altshuler, Vladimir Nesterenko, Ricardo Alfici, Ariel Halevy

**Affiliations:** 1 Trauma Unit, Division of Surgery, Assaf Harofeh Medical Center, Zerifin 70300, Israel, affiliated to the Sackler Faculty of Medicine, Tel Aviv University, Tel Aviv, Israel; 2 Trauma Unit, Division of Surgery, Hillel Yaffe Medical Center, Hadera, Israel, affiliated to the Bruce Rappoport School of Medicine, Technion, Haifa, Israel; 3 Interventional Radiology Unit, Assaf Harofeh Medical Center, Zerifin 70300, Israel, affiliated to the Sackler Faculty of Medicine, Tel Aviv University, Tel Aviv, Israel

## Abstract

**Background:**

Patients with severe pelvic fractures represent about 3% of all skeletal fractures. Hemodynamic compromise in unstable pelvic fractures is associated with arterial hemorrhage in less than 20% of patients. Angiography is an important tool in the management of severe pelvic injury, but indications and timing for its performance remain controversial.

**Methods:**

Patients with major pelvic fractures [Pelvic Abbreviated Injury Score (AIS) ≥ 3] admitted to two high volume Trauma Centers from January 2000 to June 2005 were identified and divided into two groups: Group I patients did not undergo angiography, Group II patients underwent angiography with/without embolization. Demographics, hemodynamic status on admission, concomitant injuries, Glasgow Coma Scale (GCS), Injury Severity Score (ISS), pelvic AIS, blood requirement before and after angiography, arterial blood gases and mortality were evaluated. Patients with an additional reason for hemodynamic instability were excluded.

**Results:**

Charts of 106 patients were retrospectively reviewed. Twenty nine patients (27.4%) underwent angiography. Bleeding vessel embolization was performed in 20 (18.9%) patients. Patients who underwent angiography had a significantly higher pelvic AIS and a lower Base Excess level on admission. A blood transfusion rate of greater than 0.5 unit/hour was found to be a reliable indicator for early angiography.

**Conclusion:**

A high pelvic AIS, amount of blood transfusions and decreased BE level should be considered as an indicators for early angiography in patients with severe pelvic injury.

## Background

Pelvic fractures constitute about 3% of all skeletal fractures and range in severity from low-energy stable fractures to high-energy injuries with unstable fracture patterns [1-3].

Hemodynamic compromise is not uncommon in patients suffering from unstable pelvic fracture. Bleeding is usually of venous origin. However, in 10 to 20% of the patients hemodynamic instability is associated with arterial hemorrhage [4]. Mortality of up to 50% has been reported despite effective control of bleeding [5].

Angiography is an important tool in treating arterial bleeding in hemodynamically unstable patients suffering from pelvic fractures. Indications and proper timing for performing pelvic vessel angiography remains controversial. The purpose of this retrospective study was to evaluate our experience in managing patients with severe pelvic fractures in order to clarify indicators that might help to identify those patients who may benefit from pelvic angiography and embolization.

## Methods

The study was approved by the Institutional Review Board at Assaf Harofeh Medical Center. The trauma registry was used to identify patients with major pelvic fractures, defined as Pelvic Abbreviated Injury Score (AIS) ≥ 3, admitted to two medical centers, Assaf Harofeh Medical Center (AHMC) and Hillel Yaffe Medical Center (HYMC), between January 2000 and June 2005. All patients were initially managed according to the Advanced Trauma Life Support (ATLS) protocols of the American College of Surgeons.

Patients underwent evaluation for intra-thoracic and intra-abdominal sources of hemorrhage. Pelvic radiography was routinely performed in the trauma bay according to institutional protocol. Focused abdominal sonography for trauma (FAST) was performed in each patient as part of the initial assessment. Patients who had a positive FAST result (free fluid in the peritoneal cavity) and did not response to fluid resuscitation underwent urgent laparotomy. CT of the abdomen and pelvis was performed in all stable patients. CT was performed in each patient who primarily underwent angiography due to hemodynamic compromise. Patients with hemodynamic compromise who did not respond to initial fluid resuscitation and had no source of bleeding other than the broken pelvis, and patients who had a contrast "blush" on CT underwent selective pelvic angiography along with those who had large a pelvic hematoma on non-therapeutic laparotomy. A blind embolization of iliac vessels was never performed. Internal pelvic stabilization was not carried out during the first 48 hours. In cases when an arterial bleeder was not found on angiography, the patients with an open abdomen underwent pelvic packing. All patients with hemodynamically significant bleeding from body areas other than the fractured pelvis and patients with spinal shock were excluded from the study.

Patients were then divided into two groups according to whether a therapeutic angiography (i.e. arterial bleeding treated with angiographic embolization) was performed or not. The two hospitals differ in their approach to hemodynamic compromise in patients with serious pelvic fractures. According to local protocol, at AHMC angiography is usually performed in patients who are still fluid and blood dependent following a transfusion of 2 units of blood. At HYMC, the need for and timing of angiography is individualized for each patient and is based upon the discretion of the trauma attending on call. Pelvic angiography was performed using a standard groin approach. Areas of hemorrhage were selectively embolized. No blind embolizations were performed.

Demographic and clinical variables were retrieved from the hospital charts and trauma registry. Specifically, seven variables were evaluated with respect to their relationship to whether therapeutic angiography was eventually performed or not: age at admission; initial systolic blood pressure; initial base excess; Glasgow Comma Scale on admission; bleeding rate; Pelvic AIS and ISS. Pelvic AIS was calculated based on CT results. These variables were chosen since most are readily available during the initial hours of treatment for each of these patients. We defined bleeding rate by calculating the average rate of blood units transfusion to the point when angiography was performed or during the first 24 hours in those cases where angiography was not done at all.

### Data analysis

Sensitivity, specificity, positive predictive value, negative predictive value, corresponding 95% confidence intervals, and likelihood ratios were calculated for the different variables. *P *values were calculated by using the two sided exact probability test devised by Fisher, Irwin and Yates. Analysis was performed by using statistical software (GraphPad InStat 3.06; GraphPad Software Inc, San Diego, CA).

## Results

**Figure 1:**
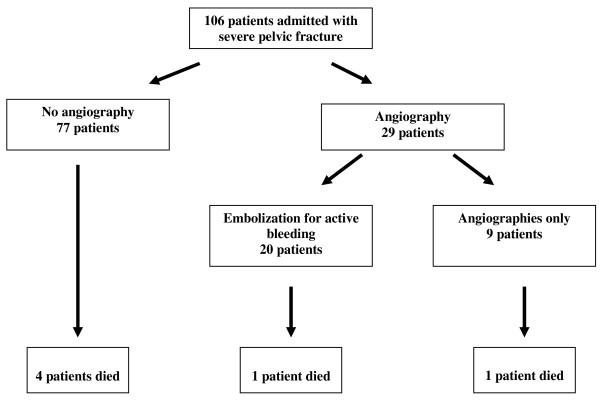
**Intervention in 106 patients admitted with significant pelvic fractures**.

One hundred and six patients with major pelvic fractures were treated at HYMC and AHMC during the study period (figure 1). Patient characteristics are presented in Table 1. The mean age was 41.3 ± 19 years. Most of the patients were male. The most common mechanism of injury leading to pelvic fracture and subsequent angiography was motor vehicle accidents. Of note is that motor vehicle accidents represent the main mechanism of injury leading to serious injury seen in both emergency departments. All patients but three suffered from associated injuries (Table 2). Six patients (5.8%) died (four from severe head injury and two from multisystem organ failure). Of these, only one patient underwent angiography. His mortality was the direct result of major head trauma. Mean time from injury to death was 3 ± 2 days. Three patients (two had a therapeutic embolization and one did not undergo angiography) developed adult respiratory distress syndrome, but survived until discharge from hospital.

**Table 1:** Patients' clinical characteristics.

Average Age (years)	41.3
Gender	
Male	70
Female	36
Mechanism of Injury	
MVA	70
PHBC	10
Fall	16
Crush Injury	6
MCC	3
Gunshot wound	1
ISS (no. of patients)	
9-14	1
16-25	37
26-50	54
51-75	14

MVC: motor vehicle accident; PHBC: pedestrian hit by car; MCC: motorcycle crash; ISS: Injury Severity Score

**Table 2:** Associated injuries in 103 patients.

Site of injury	Frequency
Chest	
Lung Contusion	27
Pneumothorax	15
Hemothorax	7
Ribs and Sternum fractures	22
Flail Chest	4
Tension Pneumothorax	3
Skeletal	
Long Bones Fracture	30
Spine Fracture	10
Head	
Brain Concussion	12
Intracerebral Hemorrhage	7
Abdomen	
Liver	9
Spleen	14
Genitourinary and/or Bowel	16

Initial stabilization of the pelvis in order to decrease venous hemorrhage was accomplished by a pelvic belt in11 (10.4%) patients. External fixation was applied in seven (6.6%) patients. Ten patients (55%) stabilized after the procedure, but eight remained unstable and proceeded to angiography (six therapeutic).

Fifteen patients (14.2%) did not undergo a CT scan of the abdomen and pelvis as part of their initial evaluation due to hemodynamic instability. Nineteen unstable patients (16.3%) underwent explorative laparotomy for suspected intraabdominal injuries. All of these patients had intrabdominal fluid based on FAST examination and seventeen were found to be suffering from a large expanding pelvic hematoma.

All angiographies were performed within 3.5 ± 2 hours following admission. An angiography was performed in the operating theater in all patients who underwent an explorative laparotomy. Overall, 29 patients (27.4%) underwent angiography. Of these, only 20 (18.9% of the patients included in the study) were diagnosed with an arterial hemorrhage that necessitated therapeutic embolization. The other nine patients, in whom an arterial bleed was not detected, will be analyzed together with the other 77 patients who did not undergo angiography.

Table 3 summarizes the likelihood with which different variables predicted those patients who would need therapeutic angiography. The transfusion rate was found to be a reliable indicator. In our patient population, a transfusion rate of beyond 0.5 units of blood per hour identified most of the patients. Hemodynamic parameters stabilized in all patients who underwent successful angioembolization and they required significantly decreased amounts of transfused blood after the procedure (9.2 ± 7.07 before vs. 2.9 ± 1.72 after, p = 0.0011). Initial base excess was another indicator found to be a predictor of therapeutic angiographies. However, utilization of this parameter on its own would have led to the performance of many other unnecessary angiographies as well. Pelvic AIS was found to be very specific. None of our patients with pelvic AIS 3 was in need of therapeutic angiography. No angiography-related complications were observed.

**Table 3:** Possible predictors of therapeutic angiography.

Possible predictors	Patients		Sensitivity (95% CI)	Specificity (95% CI)	Positive predictive value (95% CI)	Negative predictive value (95% CI)	Likelihood ratio	*P *value
							
	with therapeutic angiography	no therapeutic angiography						
**Age**								
**Age ≥ 55**	3/20	20/86	0.85	0.23	0.20	0.87	1.1	0.55
			(0.62-0.97)	(0.15-0.34)	(0.12-0.31)	(0.66-0.97)		
**Initial systolic BP**								
**< 90**	3/20	7/86	0.15	0.92	0.30	0.83	1.84	0.39
			(0.03-0.38)	(0.84-0.97)	(0.07-0.65)	(0.73-0.89)		
**Initial BE**								
**≤ -4**	15/20	24/83	0.75	0.71	0.38	0.92	2.59	0.0002
			(0.51-0.91)	(0.60-0.80)	(0.23-0.55)	(0.83-0.97)		
**≤ -6**	8/20	14/83	0.40	0.83	0.36	0.85	2.37	0.03
			(0.19-0.64)	(0.73-0.90)	(0.17-0.59)	(0.76-0.92)		
**GCS**								
**≤ 13**	10/20	26/86	0.50	0.70	0.28	0.85	1.65	0.12
			(0.27-0.73)	(0.59-0.79)	(0.14-0.45)	(0.75-0.93)		
**≤ 8**	5/20	14/86	0.25	0.84	0.26	0.83	1.54	0.35
			(0.09-0.49)	(0.74-0.91)	(0.09-0.51)	(0.73-0.90)		
**PC per hour**								
**0.5 ≤**	19/20	10/86	0.95	0.88	0.65	0.99	8.17	< 0.0001
			(0.75-1.0)	(0.80-0.94)	(0.46-0.82)	(0.93-1.0)		
**1 ≤**	15/20	7/86	0.75	0.92	0.68	0.94	9.2	< 0.0001
			(0.51-0.91)	(0.84-0.97)	(0.45-0.86)	(0.87-0.98)		
**1.5 ≤**	13/20	3/86	0.65	0.96	0.81	0.92	18.63	< 0.0001
			(0.41-0.85)	(0.90-0.99)	(0.54-0.96)	(0.85-0.97)		
**2 ≤**	11/20	3/86	0.55	0.97	0.79	0.90	15.77	< 0.0001
			(0.32-0.77)	(0.90-0.99)	(0.49-0.95)	(0.82-0.95)		
**Pelvic AIS**								
**4-5**	20/20	54/86	1.0	0.37	0.27	1.0	1.59	0.0004
			(0.83-1.0)	(0.27-0.48)	(0.17-0.39)	(0.89-1.0)		
**5**	11/20	11/86	0.55	0.87	0.50	0.89	4.3	0.0001
			(0.32-0.77)	(0.78-0.93)	(0.28-0.72)	(0.80-0.95)		
**ISS**								
**25 ≤**	17/20	61/86	0.85	0.29	0.22	0.89	1.20	0.27
			(0.62-0.97)	(0.20-0.40)	(0.13-0.33)	(0.72-0.98)		
**50 ≤**	6/20	12/86	0.30	0.86	0.33	0.84	2.15	0.10
			(0.12-0.54)	(0.77-0.93)	(0.13-0.59)	(0.75-0.91)		

BP: Blood Pressure; BE: base excess on presentation; GCS: Glasgow Coma Scale on presentation; PC: packed red blood cell units; AIS: Abbreviated Injury Score

## Discussion

Hemodynamic instability following pelvic fractures is not uncommon and is caused by disruption of the arterial and venous pelvic networks. Venous bleeding is the most common cause of hemorrhage in patients with pelvic fractures and it may be as devastating as arterial bleeds [3,6]. Pelvic stabilization is the most effective mean of controlling venous bleeding. Simple measures such as a large sheet wrapped snugly around the pelvis are thought to provide urgent pelvic stability, which in turn will allow the pelvic hematoma to organize. Whenever ongoing bleeding is caused by an arterial injury, angiography and embolization are indicated. In most series reported to date, angiographic embolization was needed in 1.9-3% of the patients admitted with pelvic fractures [7,8].

The objective of this study was to try to define criteria which would help identify those patients suffering from arterial bleeding who may benefit from angiographic embolization. It has been suggested that patients who undergo early embolization have a significantly greater survival rate [7]. Thus, early identification of trauma victims who harbor pelvic arterial bleeding has it merits.

Six of 106 in our study population died, but none as a result of exsanguination. Agolini et al. [7] reported that none of their patients died of exsanguination and most deaths were the result of multiple organ failure or severe head injury. This observation was reported by others as well [5,9]. Thus ongoing hemorrhage from a pelvic fracture, rather than being the main cause of death, acts in most cases as a contributing factor to mortality from other causes.

We defined those patients who underwent angiographic embolization as patients who had undergone therapeutic angiographies. To eliminate biases we grouped together patients who did not undergo angiography together with patients who did undergo angiography and were not found to have an arterial bleed. Evaluating different variables such as age, hemodynamic parameters and severity scores, and their association with therapeutic angiography we found that blood transfusion requirement beyond 0.5 packed red blood cell unit/hour was relatively the most efficient criteria in deciding who should undergo angiography and who should not. Using this criterion, we would have identified 19 of 20 patients who eventually needed angiography and embolization while performing ten unnecessary angiographies. Increasing the threshold to transfusion needs beyond 1 packed red blood cell unit/hour would have decreased the amount of unnecessary angiographies to seven patients. However, five patients with arterial bleeding would have been missed.

Initial base excess smaller than or equal to -4 was next in its efficacy and it would have identified 15 of 20 patients who eventually needed angiography and embolization while 24 unnecessary angiographies would have been done.

Our findings conform to those of Miller et al. and other authors who found that none of the hemodynamic parameters measured on admission (systolic blood pressure, heart rate, and base deficit) were reliable in differentiating patients who may benefit from angiography from those who will not [10,11]. It is the ongoing hemodynamic instability that best identifies patients with arterial hemorrhage [10,12]. This having been said, it is important to realize that hemodynamic stability does not rule out the need for angiography and embolization. In Miller's study, some of the patients who ultimately needed angiographic embolization did not suffer from any episodes of hypotension. Miller and his colleagues emphasize the value of performing CT angiography in stable patients since angiography performed on the basis of the presence of contrast blush, size of pelvic hematoma, or fracture pattern perceived to place the patient at high risk of arterial bleeding led to the identification of an arterial bleed and embolization in 29% of their study population.

Reviewing the literature, we found it very difficult to compare our results to those of others. The major limitation with most of the articles published to date is that inclusion criteria and presentation of data are different. Most authors chose to compare variable means of different groups using different statistical analyses. Comparing means does not allow sensitivity and specificity of different thresholds to be identified. Relying solely on P values (i.e. less than or equal to 0.05) may lead to misinterpretation of the real clinical significance of the different variables studied. For example, in our study, a prevalence of a pelvic AIS of 5 was found to be significantly higher in patients in need of embolization (p < 0.0001). However, using this criterion would have led to the recognition of only 11 (55%) of 20 patients who eventually needed this procedure, while subjecting 11 other patients to an unnecessary angiography.

Pelvic AIS was found to be a sensitive indicator for angiography. Unfortunately, the final pelvic AIS can only be precisely calculated after interpretation of the CT results. Pelvic x-ray was not found to be a sensitive indicator, missing about 1/3 of all pelvic fractures [13,14].

Another example is offered by Velmahos et al., who reported that age over 55 years was an independent predictive factor of arterial bleeding identified on angiography (p = 0.003) [15]. However, according to data from that study, if age over 55 would have served as the sole criterion for performing angiography, this would have led to the appropriate treatment of only 28% of our patients with an arterial source of bleeding.

Referral to angiography should be liberal if one wants to diagnose arterial bleeding early. Both our results and those of others indicate that none of the parameters is good enough on its own to reliably identify all the patients with an arterial bleed [15]. There are, however, several costs to a liberal policy for performing angiography: the amount of non-therapeutic angiographies will increase significantly, patients suffering from various other injuries may be subjected unnecessarily to a procedure which is both prolonged and invasive.

## Conclusion

Based on our results and those of Miller et al., we believe that patients admitted with severe pelvic fractures should undergo evaluation and resuscitation at first. Patients who are found to be stable or quickly respond to resuscitation should undergo CT angiography before subjecting them to angiography [11,16]. We believe that patients with a high pelvic AIS who are hemodynamically decompensated, and who are in continuous need of blood transfusions, should undergo angiography as early as possible. The cutoff point for this decision should be within the first hours of treatment, offering enough time to rule out and control other sources of serious bleeding.

## Competing interests

The authors declare that they have no competing interests.

## Authors' contributions

IJ has made substantial contributions to conception and design of the study, acquisition of data and analysis and interpretation of data; drafting the article and revising it critically for important intellectual content. IA has made substantial contributions to conception and design of the study, acquisition of data and analysis and interpretation of data; drafting the article and revising it critically for important intellectual content. BK has made substantial contributions to conception and design of the study and acquisition of data; and drafting the article. VG has made substantial contributions to conception and design of the study, acquisition of data and analysis and interpretation of data; and revising the article critically for important intellectual content. AP has made substantial contributions to acquisition of data and analysis and interpretation of data. AA has made substantial contributions to conception and design of the study and acquisition of data. VN has made substantial contributions to conception and design of the study and acquisition of data. RA has made substantial contributions to conception and design of the study. AH has made substantial contributions to conception and design of the study, acquisition of data and analysis and interpretation of data; and revising the article critically for important intellectual content. All authors have read and approved the final version of the manuscript.
